# Prevalence and risk factors of postpartum depression in the Middle East: a systematic review and meta–analysis

**DOI:** 10.1186/s12884-021-04016-9

**Published:** 2021-08-06

**Authors:** Hoda Alshikh Ahmad, Asem Alkhatib, Jiayou Luo

**Affiliations:** 1 Department of Maternal and Child Health, Xiangya School of Public Health, Central South University, 110 Xiangya Road, Changsha, 410078 China; 2 Department of Maternal and Child Health, Faculty of Nursing, Hama University, Hama, Syria; 3 Xiangya School of Nursing, Central South University, Changsha, China

**Keywords:** Prevalence, Risk factors, Postpartum depression, Middle East

## Abstract

**Background:**

Postpartum depression (PPD) is a common social health problem that affects not only the mother and newborn, but extends to other family members as well as various aspects of their lives. This systematic review and meta–analysis aims to identify the prevalence and risk factors of postpartum among the women in Middle East countries.

**Methods:**

We searched published articles from Web of Science, EMBASE, PubMed and Cochrane electronic databases to establish study articles. Articles regarding postpartum depression prevalence and associated factors among women in the Middle East were included in this systematic review and meta–analysis. A random–effect model was used for estimation of pooled postpartum depression prevalence with a 95% confidence interval (CI) and forest plot. Presence of heterogeneity was checked by Cochran's (Q) test, and funnel plots and Egger’s statistical tests were used to assess publication bias.

**Results:**

A total of 15 studies were included in this systematic review. The studies were conducted in different countries of the Middle East between 2006 and 2020, nine of the included studies were cross–sectional studies and six were cohort studies. The overall pooled estimate of the prevalence of postpartum depression in the Middle East mothers was very high 27% (95% CI 0.19–0.35). The common risk factors reported based on our review were poor economic, pregnancy associated complications, low education, unplanned pregnancy, housewife, inadequate social support from family members and the feeding by formula. Poor economic and complication during pregnancy presented a significant relationship regarding postpartum depression in meta–analysis.

**Conclusions:**

The prevalence of postpartum depression in the Middle East was higher than other regions of the world. In response to this, we recommend an increase of routine screening for depression during postpartum in this area. Furthermore, it might be necessary to integrate mental health with maternal health care in clinical practice during the postpartum.

**Supplementary Information:**

The online version contains supplementary material available at 10.1186/s12884-021-04016-9.

## Background

One of the challenging transition periods for mothers is the postpartum period [1], episodes of depression can be twice higher than during other periods of a woman’s life [2]. Postpartum depression (PPD) refers to any major or subclinical depression [3]. It is considered as a common social and mental health problem [4], and described as a widespread complication of child bearing. Typically, it occurs within 4–6 weeks after childbirth, but it may last several months or even a year [5]. Furthermore, up to 50% of the women will face a reoccurrence during subsequent pregnancies [6]. This illness tends to develop into major depression and may carry substantial risk to morbidity and mortality among under diagnosed situations. It has been projected that by the year 2030 if interventions are not developed as part of preventive measures, depression might be the top three leading causes of death globally [7]. Despite this, PPD is one of the least addressed types of depression today [6].

Therefore PPD is a social health burden regardless of cultural identity and beliefs [8], and it is a major public health issue which affected 10%–15% of mothers in developed societies [9]. Globally, the PPD among mothers ranges from 0.5% to 60.8% as per prevalence [10]. In recent years, it was found that PPD may affect up to 30% of all women after delivery [11], and usually ranging in severity from mild and moderate by 50–80% of women to intensive psychosis which affects less than 1% of women[12].

Due to the importance of postpartum depression and its consequences, many studies have tried to determine the level of prevalence and explore the influencing factors on PPD. In the last two decades, although large events and the status of internal and external conflicts, which increased in various the Middle East countries, studies have remained limited to understand the impact of these events on women and particularly through pregnancy, birth and postpartum phases [13, 14], in spite of studies were few however some of these studies were found as a result of the aforementioned various factors significant social, psychological and economic influencing factors which have an actual and direct impact on the postpartum phase [5, 15, 16], in addition to the traditional and widespread factors such as body image changes [17], unplanned pregnancy [18, 19], and newborn care pressures [20–25]. The situation was further complicated by the spread of the Covid–19 epidemic and the followed subsequent quarantine, which in turn led to an increase in the economic, social, and psychological burdens on societies and especially the women, in addition to increasing the pressures on medical teams, which weakens the quality of health care provided in the postpartum period [26–29].

Therefore, there was a need for a systematic review and meta–analysis, especially since it was not separately investigated in a previous study and it was only mentioned in a meta–analysis for all regions of the world, which in turn showed a higher prevalence of postpartum depression compared to other regions [30], and this increased our interest and motivated us to design this study to update the information and explore the effect of various factors on postpartum depression of the Middle East women.

## Methods

We searched our articles according to Preferred Reporting Items for Systematic review and Meta–Analyses (PRISMA) guidelines [31].

### Search strategy

To identify relevant studies strategy of three steps was used: (i) examine the targeted electronic databases systematically, (ii) examine of the included articles references manually, and (iii) discussion with an expert team. The searched electronic databases were Web of Science, EMBASE, PubMed, and Cochrane Library. We used the key MeSH search terms: such as (“mothers” or “females” or “women”), with (“postpartum” or “puerperium” or “post-birth” or “post birth”), with (“depressive” or “depression” or “PPD” or “depressed”), with (“prevalence” or “spread” or “risk factors” or “factor” or “risk” or “screening”), with (“middle east” or “Bahrain or Bahraini” or “Iraq or Iraqi” or “Iran or Iranian” or “Israel or Israeli or Jewish” or “Jordan or Jordanian” or “Kuwait or Kuwaiti” or “Lebanon or Lebanese” or “Oman or Omani” or “Palestine or Palestinian” or “Qatar or Qatari” or “Saudi Arabia or Saudi” or “Syria or Syrian” or “Turkey or Turkish” or “United Arab Emirates or Emirati” or “Yemen or Yemeni” or “Arab or Arabic”). Reference lists from pertinent articles were also screened. The title and abstract screening were independently conducted by two reviewers and identified possibly pertinent articles for the full–text review. We used Cohen kappa (K = 0.8) to assess interrater agreement. For discrepancies, we screened with the help of a third reviewer. Through discussion, articles for the full–text review were determined. This was followed by reading full texts of all articles independently to determine the final included articles in the review. We used Cohen kappa (K = 0.9) to assess inter–rater agreement.

### Selection criteria

Selection criteria of our study are the following: (a) published in English language from January 1^st^ 1990 to May 1^st^ 2020; (b) used validated assessment tools of postpartum depression while reporting the prevalence and risk factors; (c) type of studies (Cross–section and Cohort); (d) studies included women from the Middle East countries.

Studies were excluded from the review if met any of the following criteria: (1) studies did not use PPD assessment tools or with prior history of clinical symptoms; (2) included women who experienced postpartum depression more than 12 months of postpartum; (3) mothers with babies with congenital anomalies or who delivered stillborn.

### Data extraction

The parameters were extracted from the included articles as follows: name of authors, publication year, country, sample size, design, age, measure timing, and measurement of PPD, cut–off point, prevalence, and risk factors from the included studies. A standardized data extraction checklist was used for extracting the data by two reviewers and incongruities were compromised by discussion between the reviewers.

### Quality assessment

The Joanna Briggs Institute Meta–Analysis of Statistics Assessment and Review Instruments (JBI–MAStARI) for observational cohort and cross–sectional studies were used for quality assessment [32]. This assessment instruments values each study based on 11 criteria (cohort) and 8 criteria (cross–section). For each criterion, if “yes” we assigned a score of one, otherwise we assigned a score of zero score (ie, an answer of “no,” “not clear,” “not applicable,”). A total score of each study which ranging from zero to 11 (cohort) and 8 (cross–section) was computed by summing up scores of all items the instrument. Finally, studies that had scored 9 and above (cohort) or 6 and above (cross–section) on the JBI quality assessment instrument were included in the systematic review and meta–analysis. Assessment of quality assisted to measure the strength of scientific evidence only.

### Statistical analysis

Depression prevalence was reported based on self–reported assessment instrument (EPDS) to determine PPD in each study. The prevalence rate of the latest screening was used in longitudinal studies. Subgroup and meta-regression analysis were performed to investigate the effect of assessment time and the cut–off score of depression scale (EPDS) on the prevalence of PPD. Studies reported PPD in a similar period were grouped together according to assessment time points such as: (i) 0–3 months, 4–12 months. Cut–off score of depression scale (EPDS) 10, 12, and 13 were used to compare the prevalence with different depressive symptoms. A funnel plot, Egger and Begg’s statistical tests were employed to test the presence and the statistical significance of publication bias. The meta–analysis was conducted by Version 5.4 of Review Manager software and meta-regression analysis was conducted by Version 17 of STATA software. Cochran's Q–test (Chi^2^) and Quantitative heterogeneity were explored using I^2^ statistics. The I^2^ statistics refers to the variance percentage between studies to heterogeneity rather than sampling chance [33]. The assessment of heterogeneity (I^2^) was considered low, moderate, and high heterogeneity, corresponding to 25%, 50%, and 75% respectively [33]. The random–effects model was used when heterogeneity was moderate or high (I^2^ > 50%) to originate the overall prevalence estimate. For each forest plot, the combined estimates heterogeneity test values (T^2^, Chi^2^, and I^2^), Z–value, and 95% CI were reported. For factors associated with PPD, a random–effects model also was used to pool the odds ratios among included studies.

## Results

### Study selection

**Fig. 1:**
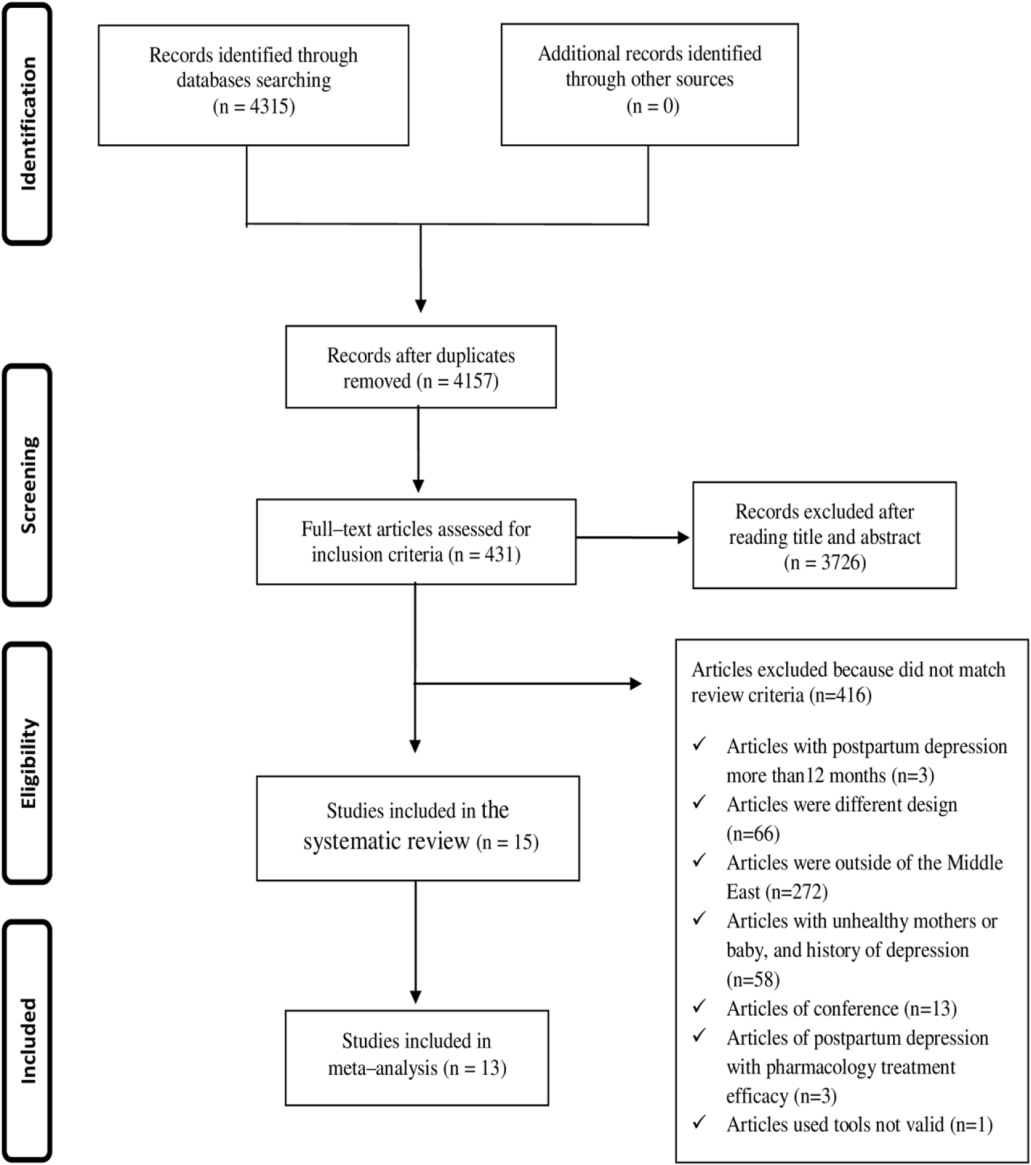
PRISMA flowchart of study selection

Initially, 4315 studies were accessed from the searched electronic databases, of which 4157 remained after removing duplicates. After reading title abstracts, 431 studies were included for full–text review, of which 15 studies met all the inclusion criteria in this systematic review. The PRISMA flow chart for the selection procedures and the reasons for exclusion were shown in Fig. 1.

### Overview of included studies in the systematic review

In our study, we presented the major characteristics of the included studies in this systematic review (See Additional File 1). They were conducted in different countries of the Middle East, five of them were conducted in Iran, two in Israel, two in Turkey, two in the United Arab Emirates, one in Jordan, one in Oman, one in Qatar. All of the articles included in this study were published between 2006 and 2020. Of the 15 articles included in the systematic review, six were cohort studies and nine were cross–sectional studies. Covering a total of 6683 women in the period of postpartum, the sample size ranges from a minimum of 56 in the United Arab Emirates to a maximum of 1379 in Qatar.

In a current meta–analysis, to estimate the pooled prevalence of postpartum depression in the Middle East, 6074 women in the postpartum period from 13 studies were included, two papers were excluded from meta–analysis: Iranpour et al. [34] used the same dataset as the other paper [35], and Ozgur et al. [36], only used BDI scale for depression evaluation, while all studies included in the meta–analysis were used EPDS scale.

### The prevalence of postpartum depression

**Fig. 2:**
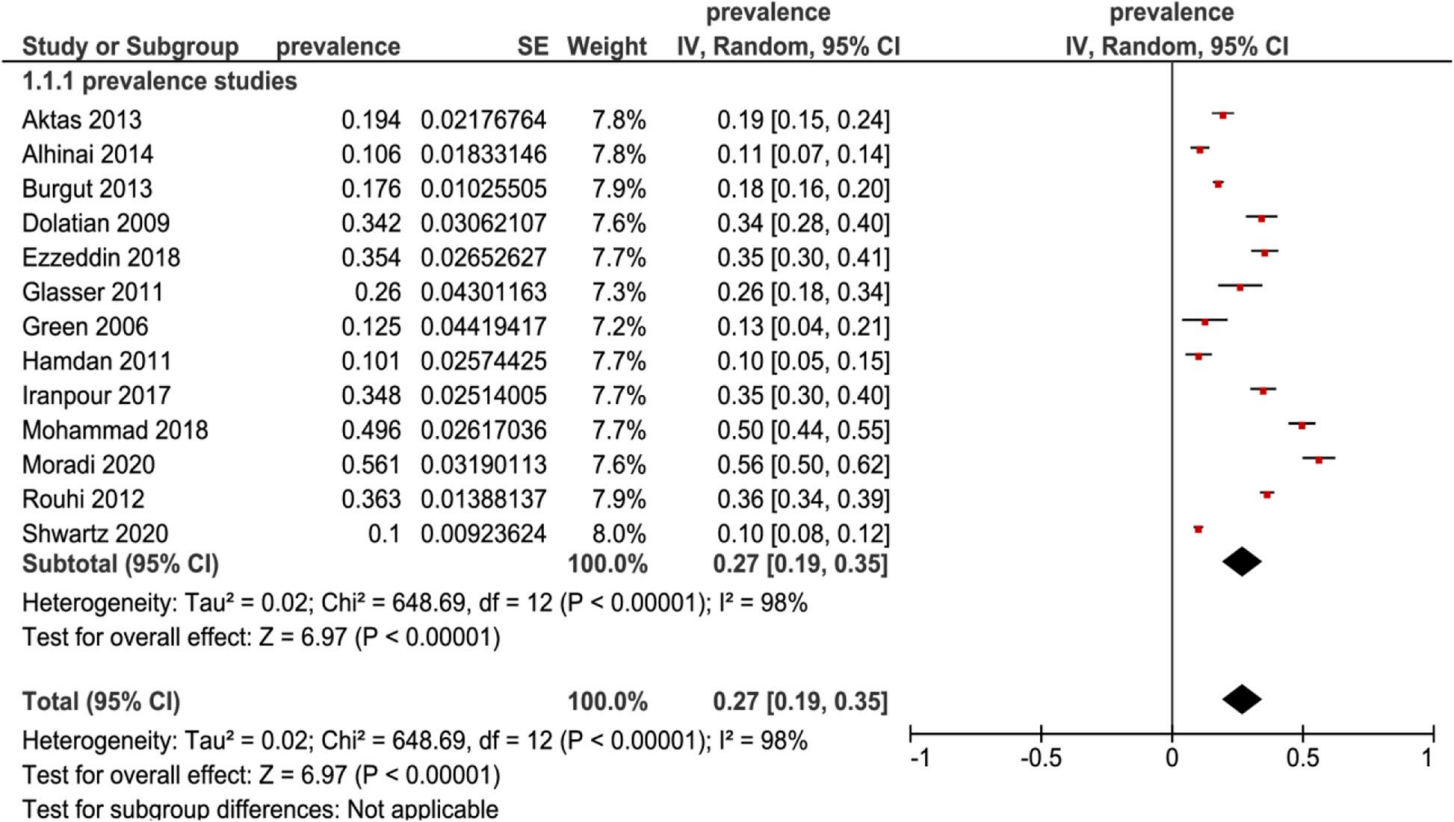
Forest plot of 13 studies assessing the prevalence of postpartum depression in Middle East

Random–effects model was used, the overall pooled estimate of the postpartum depression prevalence in the Middle East women was 27% (95% CI 0.19–0.35) Fig. 2, shows the results of 13 studies that stated the PPD prevalence.

**Fig. 3:**
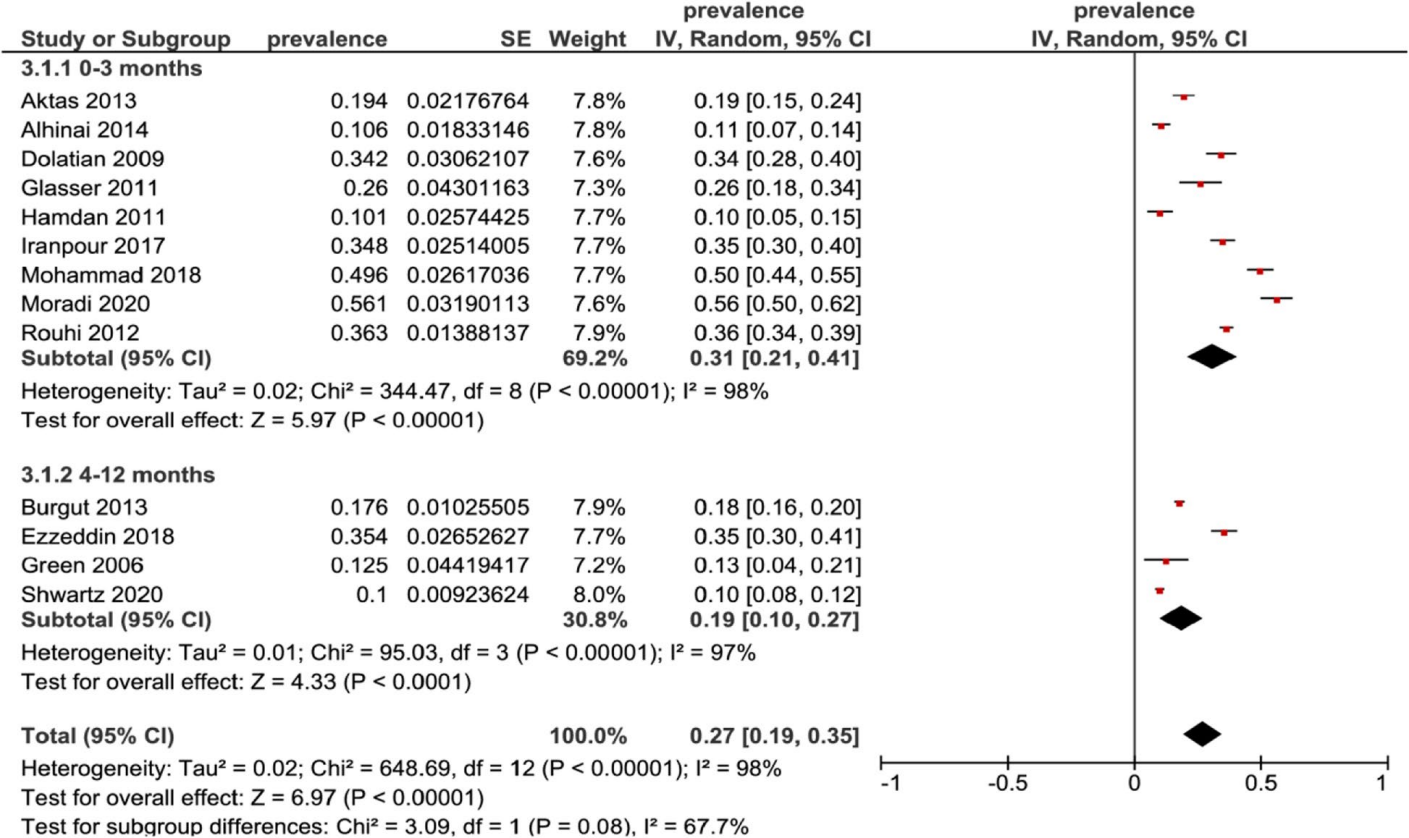
Subgroup analysis for prevalence of postpartum depression in Middle East among mothers at different assessment time points

**Fig. 4:**
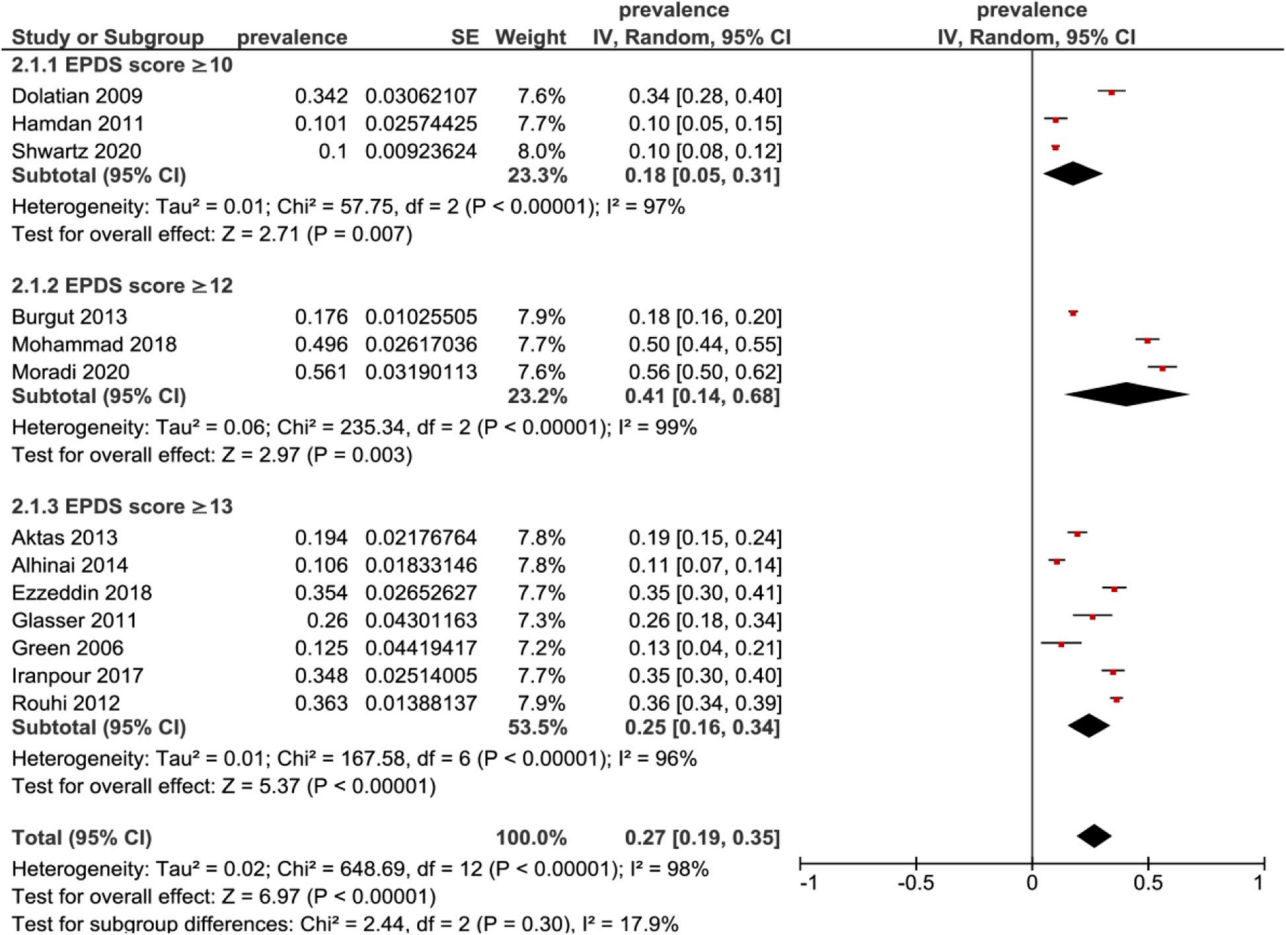
Subgroup analysis for prevalence of postpartum depression in Middle East symptoms by EPDS cut–off scores

Subgroup analysis was performed to assess the depression by time points, we had 9 studies reporting on the prevalence in the first three months of postpartum, the prevalence was 31% (95% CI 0.21–0.41, N = 3259) and 4 studies reported the prevalence from the fourth to twelfth months of postpartum, the prevalence was 19% (95% CI 0.10–0.27, N = 2815), while the overall test for time points subgroup was not significant (p = 0.08) Fig. 3. All included studies used Edinburgh postnatal depression scale (EPDS) for assessing depressive symptoms. Using an EPDS score ≥ 10 for our subgroup analysis of studies, the postpartum depression prevalence was 18% (95% CI 0.05–0.31, N = 1432), in a subgroup analysis of score ≥ 12 on EPDS, the prevalence was 41% (95% CI 0.14–0.68, N = 1986) and in a subgroup analysis of score ≥ 13 on EPDS, the prevalence was 25% (95% CI 0.16–0.34, N = 2656), while the overall test for cut–off scores subgroup was not significant (P = 0.30) Fig. 4.

Meta-regression was used to determine the potential sources of heterogeneity by using assessment time points (Coef = -0.11, SE = 0.08, P = 0.18, CI -0.29 to 0.05) and cut–off scores (Coef = 0.02, SE = 0.03, P = 0.55, CI -0.04 to 0.09), but none of these variables were statistically significant.

**Fig. 5:**
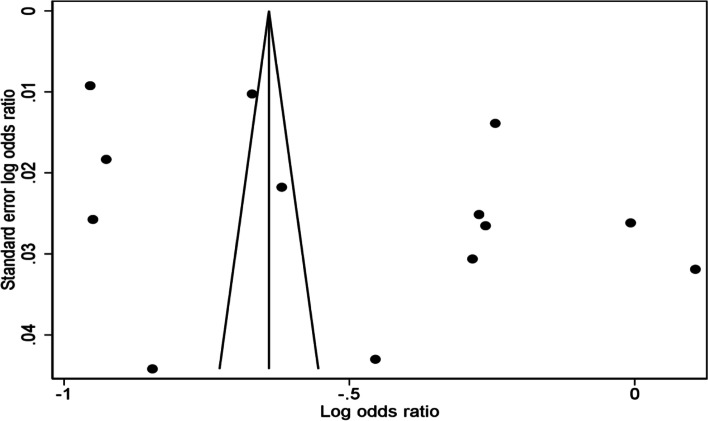
Funnel plots of publication bias

A funnel plot was used to examine the publication bias. The slight asymmetry indicates possible publication bias based on the visual representation of the funnel plot Fig. 5. In addition, Egger’s and Begg’s tests showed no statistically significant of publication bias (P = 0.457, P = 0.502), respectively.

### Risk factors of postpartum depression

A total of 15 studies reported risk factors for postpartum depression, similar factors searched in each of the studies. A total of 30 risk factors were obtained to be investigated in the included studies, and these were classified into four categories: (1)Socio–demographic factors were included low education (in 6 out of 14 studies that included this variable), work or did not work outside home (2/5/14 studies), poor economic (5/11studies) and young age (3/12 studies); (2)Marital and family relationship factors were included lack of relationship with family (5/6 studies) and lack of support from family (4/4 studies); (3)Pregnancy and birth related factors were included complications of birth and pregnancy (1/4/7 studies), planned or unplanned pregnancy (1/6/10 studies) and vaginal or caesarean Section (1/2/8 studies); (4)Newborn–related factors were included feeding by formula (5/6 studies), gender of baby (2/9 studies), unhealthy baby (2/3 studies) and difficulties caring of baby (2/2 studies), the other factors were illustrated in Table. 1.

**Table 1:** Risk factors for postpartum depression reported by studies included in the systematic review and meta–analysis

Variables		No. of studies	
		**Reporting risk for postpartum depression**	**Total**
**Socio–demographic factors**			
Age	Young age	3	12
Age at marriage	Older at marriage	1	2
Education	Low education	6	14
Economic	Poor economic	5	11
Employment & occupation	Not work outside the home	5	14
	Work outside the home	2	
Religion	Non–Muslim	1	1
Number of children	Having more than one child	1	2
Sleep quality	Low sleep quality, experienced sleep disturbances, used medication, have dysfunction	1	1
Caffeine consumption	High intake caffeine(g) and refined grain (g)	1	1
Ethnicity	Arab	1	1
Household food security status	Food insecurity increased PPD	1	1
Body image and weight	Having a negative body and Weight image at 3 months	1	2
Transportation access	Lack of access	1	1
Length of residency (immigration)	Less than 2 years	1	1
Planned to back to work after maternity leave	Not planned	1	1
Health insurance	Without health insurance	1	1
**Marital & family relationship factors**			
Relationship with family	Lack of relationship	5	6
Consanguinity	Consanguinity	1	2
Violence	Violated women	1	2
	Intimate partner violence	1	
Family support	Lack of support	4	4
History of family depression	Family with history	1	1
**Pregnancy & birth related factors**			
Planned and unplanned pregnancy	Unplanned pregnancy	6	10
	Planned pregnancy	1	
Maternal complications	Complications of pregnancy	4	7
	Complications of birth	1	
Type of delivery	Caesarean section	2	8
	Vaginal section	1	
Primigravida		1	1
**Newborn–related factors**			
Gender of baby		2	9
Health of baby	Un healthy	2	3
Type of feeding	Feeding by formula	5	6
Birth of first child		1	1
Care of baby	Difficulties caring of baby	2	2

In meta–analysis, two factors, i.e. poor economic Fig. 6, and complication during pregnancy Fig. 7, showed significant association with PPD, while the rest of the factors showed insignificant associations with PPD (Supplemental Figs. 8–15, Additional File 2).

**Fig. 6:**
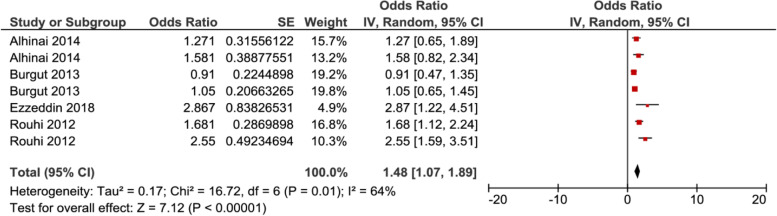
Forest plot for pooled association between Poor Economic (Ref. Economic) and postpartum depression in Middle East

**Fig. 7:**
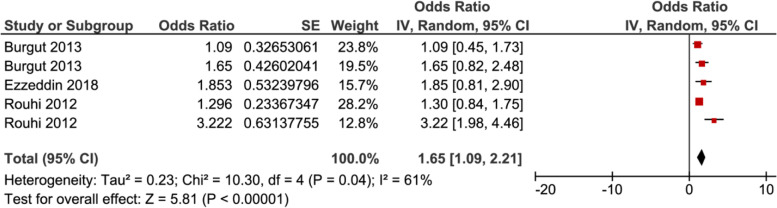
Forest plot for pooled association between Complication during pregnancy and postpartum depression in Middle East

Seven studies were included in analysis to determine the association between poor economic and postpartum depression, of which three of them showed a significant association. The pooled analysis showed that the odds of developing postpartum depression among women who had poor economic was 1.48 (95% CI 1.07–1.89) times higher as compared with their counterparts, as depicted in Fig. 6. While five studies were included to determine the association between complication during pregnancy and postpartum depression, of which two of them showed a significant association. The pooled analysis showed that the odds of developing postpartum depression among women who complication during pregnancy was 1.65 (95% CI 1.09–2.21) times higher as compared with women without complications Fig. 7.

## Discussion

In our study, we conducted a systematic review with a meta–analysis among published articles between 2006 and 2020. A total number of 15 studies met all the inclusion criteria, and nine of which are cross–sectional studies and the rest are cohort studies. The overall pooled estimated the postpartum depression prevalence in the Middle East mothers was 27%. Nine studies have reported the prevalence in the first 3 months of postpartum, and the rest reported during 4–12 months of postpartum. All the included studies in our meta–analysis used EPDS for assessing depressive symptoms. In the subgroup analysis of studies with different EPDS score cut–offs, including 10, 12, and 13, only 3 studies are found to be in the subgroup with EPDS score ≥ 12 and has the highest prevalence was compared to the other 2 subgroups.

From our meta–analysis, we found that the overall prevalence of PPD among the Middle East women is 27%. Apparently, this estimate is consistent with the previously studied estimates of 26% [30]. The reported estimate of PPD prevalence was higher than other studies in western countries, for instance the prevalence of PPD in North America was 10%–15% and in Australia was 13% [5, 37]. These statistics established that the prevalence of PPD was a higher in the Middle East than other regions of the world, the predicted reasons that lead to the wide range of reported PPD might be due to the disorders and displacement during crises and to the cross cultural variation, socio–economic conditions which might include actual or perceived levels of social support, stressful life events, poverty and attitudes towards pregnancy and motherhood at large [13, 14]. Apparently, inadequate necessary mental care, when societies do not prioritize mental difficulties for women during postpartum is also a factor [38]. On subgroup analysis, the prevalence was generated based on time of assessment. These were consistent across the first postpartum year, whereas we found a higher level of prevalence was found in the first 3 months of postpartum while a lower level reported in 4–12 month of postpartum. There were 4 studies only that examined prevalence after the third month of postpartum, compared to 9 studies that examined prevalence in the first 3 months of postpartum. Literature indicates that onset of PPD is a high–risk timeframe during the first three months postpartum [39, 40]. This could be the cause that numerous individual reports examined the prevalence of PPD in the early postpartum period only first three months [2, 39–41]. However, there are some longitudinal investigations that announced insistence in depressive symptoms a year postpartum and even more than year [42–44].

Several socio–demographic risk factors were reported for PPD. Linking poverty to inadequate education, which in turn leads to early age marriage, may affect the socioeconomic status. We found a higher proportion of postpartum depression in mothers who were younger compared with those old aged, this could be due to tender age as a mother with inadequate experience for caring of newborn or to cope with depression, therefore is more prone to show depressive symptoms in the postpartum [45], these results were consistent with results of Rich–Edwards and Inandi [46] who found that women in the youngest age group exhibited almost twice the rate of depression scales when compared to the other age groups. Housewives with lower incomes were more likely to score higher regarding EPDS, and consistent results of postpartum depression rates were showed by Taşdemir et al. and Gumuş et al. [47, 48]. The most pressing issue for housewives are poverty, these were the conclusion drawn from a study done by Denins et al. [45]. They believed that this could increase the financial stressors and perhaps pivotal to elevated depression odds. Undoubtedly, the level of education as a risk factor affects the prevalence of PPD [49]. For instance, a decrease in socio–economic status is a result of education. Conversely, a good level of education confers intellectual skills and of course advanced copying strategies. This is evident from our study that women with lower levels of education exhibited higher depressive symptoms [47].

Indeed, the transition into motherhood is complex and stressful moment in life [50]. To help ease the stress of pregnancy and motherhood, a supportive social network such as family, partner, and friends are crucial [51]. Wherefore, PPD in our study was correlated with lack of social support and observed through the literature increased risk of PPD significantly with women who reported limited support [52]. Among the factors that can complicate the transition to motherhood is unplanned pregnancy. In our study, we identified unplanned pregnancy as a significant risk factor for PPD. We predicted this as unwanted pregnancy makes it difficult to adapt to the roles and responsibilities of motherhood. Thus, it may cause the women to experience severe mental issues during the postpartum period. Consistent with previous studies designed to assess PPD risk factors, unplanned pregnancy is found to be a common risk factor [53–55]. Also, we found pregnancy complications to be a significant factor to develop PPD. For the fact that the mother’s body undergoes series of changes during the process of gestation and parturition, the presence of complication events may work as a trigger for developing depression. This aggravated the workload on the mother in addition to the maternal responsibilities and challenges. Similar to our findings was that the impact of pregnancy complications on developing PPD [56, 57].

Insufficient paediatric care as paediatric problems in infants is associated with PPD in mothers and this in turn is associated with issues related to the child. With stressful newborn–related events an increased PPD risk was observed. These include health problems and infant hospitalization [58]. Mothers who have recently given birth have trouble caring for the baby independently in early times [59], thus, identified that odds of developing PPD in the women who stated they had trouble in baby care or experienced stressful events related to newborn were higher than those of women expressing no problems. In the context of paediatric issues, nutrition of newborn consider as an essential psychological and physiological function of motherhood, perhaps consistent with this theory; mothers who did not breast feed and did formula feeding alone had an increased risk of PPD, probably due to the formula feeding reduction of the mother–infant relationship. In our study, formula feeding is a risk factor for PPD. In a similar study, women with a higher level of breastfeeding self–efficacy were seen to exhibit fewer postpartum depressive symptoms [60].

The limitations in this review included the following aspects. First, only articles published in the English language were included, so we might miss a few articles. Also, all the studies used the EPDS scale while only one study used BDI scale, so we were not able to compare the difference of prevalence between the two scales. Finally, some risk factors were only reported by one study, thus we were not able to generate the pooled effect size.

## Conclusions

In this systematic review and meta–analysis, we found the prevalence of PPD in the Middle East was determined to be 27%. Of all the 15 studies included, they were conducted in different countries of the Middle East, majority of which are 9 cross–sectional studies and 6 cohort studies. The common risk factors found to be reported based on our review are low education, unplanned pregnancy, housewife or domestic responsibilities, poor wealth index, lack of relationship with family, the feeding method of formula milk, inadequate social support from family members, young age, pregnancy associated complications, gender of baby, type of delivery, health of baby, and care of baby. Lack of access to effective postpartum care may contribute to the majority of PPD causes. Therefore, we recommend combined mental health care alongside maternal care services during the postpartum period. Providers of maternal health care need to be aware of and receive suitable guidance on psychological issues when providing care for pregnant and delivered women. The result gathered from our study can serve as evidence that can be used to guide future studies and health policies. Sensitization about the importance of screening for the PPD may help to prevent or identify and treat PPD.

## Supplementary Information

**Additional file 1.** Table of features of the studies included in the systematic review [61–70].

**Additional file 2.** Supplement Additional Figures.

## Data Availability

All data generated or analyzed during this study are included in this article (and its supplementary files).

## Abbreviations

PPD: Postpartum Depression
EPDS: Edinburgh Postnatal Depression Scale
BDI: Beck Depression Inventory
JBI: Joanna Briggs Institute
CI: Confidence Interval
OR: Odds Ratio

## References

[1] Vesga-López O, Blanco C, Keyes K, Olfson M, Grant BF, Hasin DS (2008). Psychiatric Disorders in Pregnant and Postpartum Women in the United States. Arch Gen Psychiatry.

[2] Cox JL, Murray D,Chapman G. A controlled study of the onset, duration and prevalence of. Brit J Psychiatry 1993;163(27â):31. 10.1192/bjp.163.1.27 8353695

[3] O'hara MW, McCabe JE. Postpartum Depression: Current Status and Future Directions. Ann Review Clin Psychol 2013;9(1):379–407. 10.1146/annurev-clinpsy-050212-185612 23394227

[4] Özcan NK, Boyacıoğlu NE, Dinç H (2017). Postpartum depression prevalence and risk factors in Turkey: A systematic review and meta-analysis. Arch Psychiatr Nurs.

[5] O'hara MW, Swain AM. Rates and risk of postpartum depression—a meta-analysis. Int Review Psychiatry 1996;8(1):37–54.

[6] Nonacs R. Post psychiatric syndrome. Kaplan and Sadock's Comprehensive Textbook of Psychiatry 2000.

[7] Mathers C, Loncar D. Projections of Global Mortality and Burden of Disease from 2002 to 2030. PLoS Medicine 2006;3(11):e442. 10.1371/journal.pmed.0030442 PMC1664601 17132052

[8] Evagorou O, Arvaniti A, Samakouri M (2016). Cross-cultural approach of postpartum depression: manifestation, practices applied, risk factors and therapeutic interventions. Psychiatr Q.

[9] Pearlstein T, Howard M, Salisbury A, Zlotnick C (2009). Postpartum depression. Am J Obstet Gynecol.

[10] Halbreich U, Karkun S (2006). Cross-cultural and social diversity of prevalence of postpartum depression and depressive symptoms. J Affect Disord.

[11] Evins GG, Theofrastous JP, Galvin SL (2000). Postpartum depression: A comparison of screening and routine clinical evaluation. Am J Obstet Gynecol.

[12] Stowe ZN, Nemeroff CB (1995). Women at risk for postpartum-onset major depression. Am J Obstet Gynecol.

[13] Mohammad KI, Abu Awad D, Creedy DK, Gamble J (2018). Postpartum depression symptoms among Syrian refugee women living in Jordan. Res Nurs Health.

[14] Roumieh M, Bashour H, Kharouf M, Chaikha S (2019). Prevalence and risk factors for postpartum depression among women seen at Primary Health Care Centres in Damascus. BMC Pregnancy Childbirth.

[15] Beck CT (2001). Predictors of postpartum depression: an update. Nurs Res.

[16] James-Hawkins L, Shaltout E, Abdi-Nur A, Nasrallah C, Qutteina Y, Abdul Rahim HF (2019). Human and economic resources for empowerment and pregnancy-related mental health in the Arab Middle East: A systematic review. Arch Womens Ment Health.

[17] Zaheri F, Nasab LH, Ranaei F, Shahoei R (2017). The relationship between quality of life after childbirth and the childbirth method in nulliparous women referred to healthcare centers in Sanandaj. Iran Electronic Physician.

[18] Ahmad NA, Silim UA, Rosman A, Mohamed M, Chan YY, Kasim NM, et al. Postnatal depression and intimate partner violence: a nationwide clinic-based cross-sectional study in Malaysia. BMJ Open 2018;8(5). 10.1136/bmjopen-2017-020649 PMC5961592 29764882

[19] Azad R, Fahmi R, Shrestha S, Joshi H, Hasan M, Khan A, et al. Prevalence and risk factors of postpartum depression within one year after birth in urban slums of Dhaka, Bangladesh. PloS one 2019; 14(5): p. e0215735. 10.1371/journal.pone.0215735 PMC6497249 31048832

[20] Corey E, Thapa S (2011). Postpartum depression: An overview of treatment and prevention.

[21] Dennis CL (2005). Psychosocial and psychological interventions for prevention of postnatal depression: systematic review. BMJ.

[22] Afshari P, Tadayon M, Abedi P, Yazdizadeh S (2020). Prevalence and related factors of postpartum depression among reproductive aged women in Ahvaz. Iran Health care for women international.

[23] Rai S, Pathak A, Sharma I (2015). Postpartum psychiatric disorders: Early diagnosis and management. Indian J Psychiatry.

[24] Al Nasr RS, Altharwi K, Derbah MS, Gharibo SO, Fallatah SA, Alotaibi SG, et al. Prevalence and predictors of postpartum depression in Riyadh, Saudi Arabia: A cross sectional study. PloS one 2020;15(2):e0228666. 10.1371/journal.pone.0228666 PMC7010279 32040495

[25] Norhayati MN, Nik Hazlina NH, Asrenee AR, Wan Emilind WM (2015). Magnitude and risk factors for postpartum symptoms: A literature review. J Affect Disord.

[26] Li C, Huo L, Wang R, Qig F, Wang W, Zhou X (2021). The prevalence and risk factors of depression in prenatal and postnatal women in China with the outbreak of Corona Virus Disease 2019. J Affect Disord.

[27] Liang P, Wang Y, Shi S, Liu Y, Xiong R. Prevalence and factors associated with postpartum depression during the COVID-19 pandemic among women in Guangzhou, China: a cross-sectional study. BMC Psychiatry 2020;20(1). 10.1186/s12888-020-02969-3 PMC7686811 33238927

[28] Zeng X, Li W, Sun H, Luo X, Garg S, Liu T, et al. Mental health outcomes in perinatal women during the remission phase of COVID-19 in China. Frontiers in psychiatry 2020;11. 10.3389/fpsyt.2020.571876 PMC7573142 33132935

[29] Duran ES, Dang D, Ogburn T. Comparing the trends of postpartum depression screening scores during and before the COVID-19 pandemic 2021; MEDI 9331 Scholarly Activities Clinical Years.

[30] Shorey S, Chee CY, Debby Ng, Chan YH, Tam WW, Chong YS. Prevalence and incidence of postpartum depression among healthy mothers: a systematic review and meta-analysis. J Psychiatric Res 2018;104:235–48. 10.1016/j.jpsychires.2018.08.001 30114665

[31] Liberati A, Altman D, Tetzlaff J, Mulrow C, Gøtzsche P, Ioannidis J (2009). The PRISMA statement for reporting systematic reviews and meta-analyses of studies that evaluate health care interventions: explanation and elaboration. J Clin Epidemiol.

[32] Moola S, Munn Z, Tufanaru C, Aromataris E, Sears K, Sfetcu R, et al. Chapter 7: Systematic reviews of etiology and risk. Joanna Briggs Institute Reviewer's Manual. The Joanna Briggs Institute 2017;5.

[33] Higgins JP, Thompson SG, Decks JJ, Altman DG (2003). Measuring inconsistency in meta-analyses. BMJ.

[34] Iranpour S, Kheirabadi G, Esmaillzadeh A, Heidari-Beni M, Maracy M. Association between sleep quality and postpartum depression. J Res Med Sci. 2016;21. 10.4103/1735-1995.193500 PMC5322694 28250787

[35] Iranpour S, Kheirabadi G, Esmaillzadeh A, Heidari-Beni M, Maracy M (2017). Association between caffeine consumption during pregnancy and postpartum depression: a population-based study. J Caffeine Res.

[36] Ozgur G, Atan SU, Ardahan M (2012). Postpartum depression among working and non-working women in Denizli. Turkey Healthmed.

[37] Leitch S. Postpartum depression: A review of the literature. St. Thomas, Ontario: Elgin-St. Thomas Health Unit, 2002.

[38] Baxter AJ, Patton G, Scott KM, Degenhardt L, Harvey A. Whitefordet H. Global epidemiology of mental disorders: what are we missing? PloS one 2013;8(6):e65514. 10.1371/journal.pone.0065514 PMC3691161 23826081

[39] Cooper PJ, Campbell EA, Kennerley H, Bond A (1988). Non-psychotic psychiatric disorder after childbirth: a prospective study of prevalence, incidence, course and nature. Br J Psychiatry.

[40] O’Hara MW, Neunaber DJ, Zekoski EM. Prospective study of postpartum depression: prevalence, course, and predictive factors. J Abnorm Psychol. 1984;93(2):158. 10.1037//0021-843x.93.2.158 6725749

[41] Andrews-Fike C (1999). A review of postpartum depression. Primary Care Companion J Clin Psychiatry.

[42] Chang HP, Chen JY, Huang YH, Tyan JY, Yeh CJ, Su PH (2014). Prevalence and factors associated with depressive symptoms in mothers with infants or toddlers. Pediatr Neonatol.

[43] Small R, Lumley J, Yelland J (2003). Cross-cultural experiences of maternal depression: associations and contributing factors for Vietnamese, Turkish and Filipino immigrant women in Victoria Australia. Ethnicity Health.

[44] Woolhouse H, Gartland D, Mensah F, Brown SJ. Maternal depression from early pregnancy to 4 years postpartum in a prospective pregnancy cohort study: implications for primary health care. BJOG. 2015;122(3):312–21. 10.1111/1471-0528.12837 24844913

[45] Dennis CL, Janssen PA, Singer J (2004). Identifying women at-risk for postpartum depression in the immediate postpartum period. Acta Psychiatr Scand.

[46] Rich-Edwards JW, Kleinman K, Abrams A, Harlow B, McLaughlin T, Joffe H (2006). Sociodemographic predictors of antenatal and postpartum depressive symptoms among women in a medical group practice. J Epidemiol Community Health.

[47] Gümüş A, Keskin G, Alp N, Özyar S, Karsak A (2012). The prevalence of postpartum depression and associated variables. New Sympos J.

[48] Taşdemir S, Kaplan S, Bahar A (2006). Determination of the affecting factors of postpartum depression. Fırat Sağlık Hizmetleri Dergisi.

[49] Mills E. Depression, anxiety and childbirth depression. Anxiety & Childbirth http://www.pndsa.co.za/research.htm.(30 Ekim 2008’de ulaşıldı).

[50] DeVito J (2010). How adolescent mothers feel about becoming a parent. J Perinat Educ.

[51] Watts M, Liamputtong P, Mcmichael C (2015). Early motherhood: A qualitative study exploring the experiences of African Australian teenage mothers in greater Melbourne Australia. BMC Public Health.

[52] Mills E, Finchilescu G, Lea S (1995). Postnatal depression-An examination of psychosocial factors. S Afr Med J.

[53] Glasser S, Stoski E, Kneler V, Magnezi R (2011). Postpartum depression among Israeli Bedouin women. Arch Womens Ment Health.

[54] Prost A, Lakshminarayana R, Nair N, Tripathy P, Copas A, Mahapatra R (2012). Predictors of maternal psychological distress in rural India: A cross-sectional community-based study. J Affect Disord.

[55] Caropreso L, Cardoso T, Eltayebani M, Frey B. Preeclampsia as a risk factor for postpartum depression and psychosis: A systematic review and meta-analysis. Archives Women's Mental Health 2019. p. 1–13. 10.1007/s00737-019-01010-1 31802249

[56] Blom EA, Jansen PW, Verhulst FC, Hofman A, Raat H, Jaddoe V, et al. Perinatal complications increase the risk of postpartum depression. The Generation R Study. BJOG 2010;117(11):1390–8. 10.1111/j.1471-0528.2010.02660.x 20682022

[57] Shivalli S, Gururaj N. Postnatal depression among rural women in South India: do socio-demographic, obstetric and pregnancy outcome have a role to play? PLoS One 2015;10(4):e0122079. 10.1371/journal.pone.0122079 PMC4388688 25848761

[58] Altshuler LL, Hendrick V, Cohen LS (1998). Course of mood and anxiety disorders during pregnancy and the postpartum period. J Clin Psychiatry.

[59] Beydag K (2007). Adaptation to motherhood in the postpartum period and the nurse’s role. TAF Prev Med Bull.

[60] Haga SM, Ulleberg P, Slinning K, Kraft P, Steen T, Staff A (2012). A longitudinal study of postpartum depressive symptoms: multilevel growth curve analyses of emotion regulation strategies, breastfeeding self-efficacy, and social support. Arch Womens Ment Health.

[61] Green K, Broome H, Mirabella J (2006). Postnatal depression among mothers in the United Arab Emirates: socio-cultural and physical factors. Psychol Health Med.

[62] Dolatian M, Hesami K, Shams J, Majd H (2010). Relationship between violence during pregnancy and postpartum depression. Iran Red Crescent Med J.

[63] Hamdan A, Tamim H (2011). Psychosocial risk and protective factors for postpartum depression in the United Arab Emirates. Arch Womens Ment Health.

[64] Aktaş D, Terzioğlu F (2013). Occurrence of depression during the postpartum period and risk factors that affect the development of the depression. Turkish J Med Sci.

[65] Al Hinai FI, Al Hinai SS (2014). Prospective study on prevalence and risk factors of postpartum depression in Al-dakhliya governorate in oman. Oman Med J.

[66] Rouhi M, Yousefi H (2012). Ethnicity as a risk factor for postpartum depression. Br J Midwifery.

[67] Burgut F, Bener A, Ghuloum S, Sheikh J (2013). A study of postpartum depression and maternal risk factors in Qatar. J Psychosom Obstet Gynecol.

[68] Ezzeddin N, Jahanihashemi H, Zavoshy R, Noroozi M (2018). The prevalence of postpartum depression and its association with food insecurity among mothers referring to community health centers. Iran J Psychiatry.

[69] Moradi F, Azami H, Hemmatpour B, Jamasbi M, Farahmand N, kermani S, et al. Factors Related to Postpartum Depression in Mothers Referred to Kermanshah Health Centers, Iran. J Clin Diagnostic Res 2020;14(5).

[70] Shwartz N, O’Rourke N, Daoud N. Pathways linking intimate partner violence and postpartum depression among Jewish and Arab women in Israel. J Interpersonal Violence 2020; p. 0886260520908022. 10.1177/0886260520908022 32167400

