# Supplementary material for: Prevalence and risk factors of postpartum depression in the Middle East: a systematic review and meta–analysis
Source: BMC Pregnancy Childbirth. 2021 Aug 6;21:542. doi: 10.1186/s12884-021-04016-9 (PMC8343347; doi:10.1186/s12884-021-04016-9)
Supplement: Supplementary file 2 — Additional file 2. Supplement Additional Figures. [file 12884_2021_4016_MOESM2_ESM.doc]

**Supplement Additional Figures**


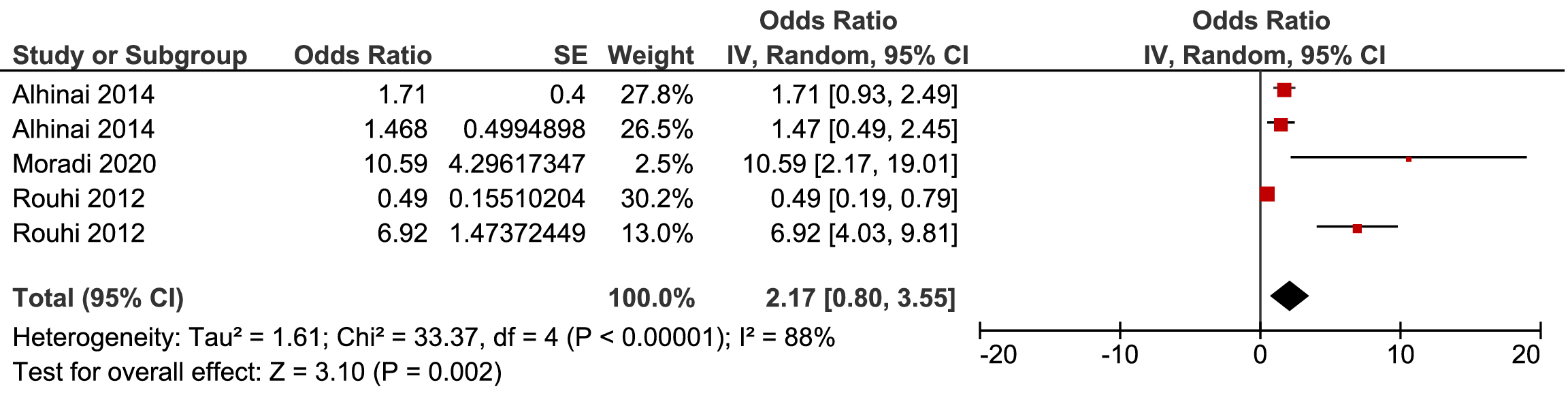


**Additional figure 8**: Forest plot for pooled association between lack of relationship with family (Ref. Relationship with family) and postpartum depression in the Middle East.


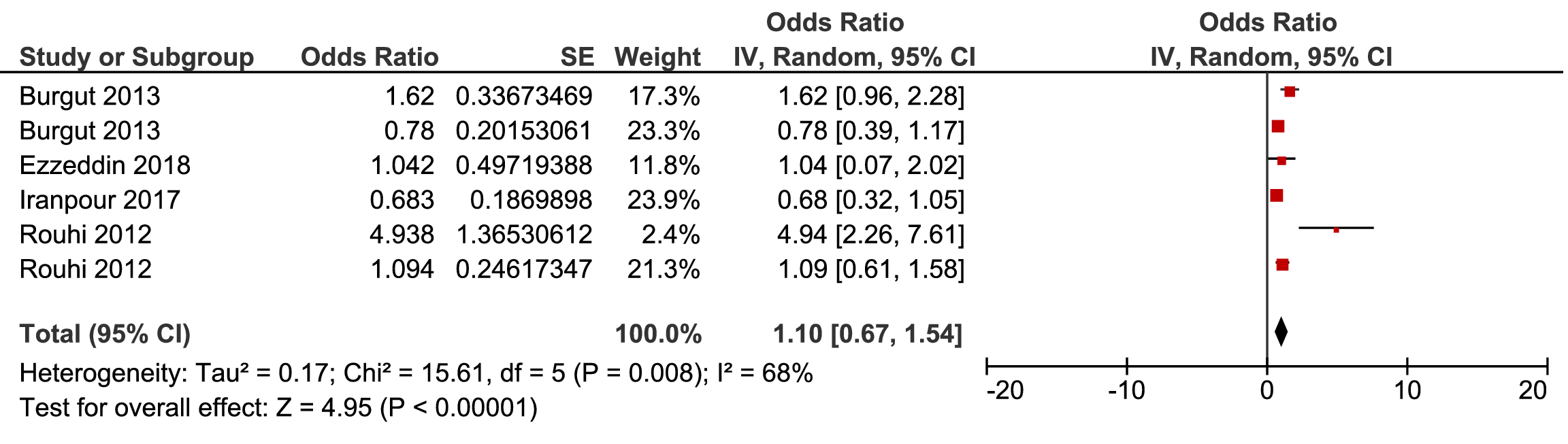


**Additional figure** **9**: Forest plot for pooled association between low education (Ref. Mother’s education) and postpartum depression in the Middle East.


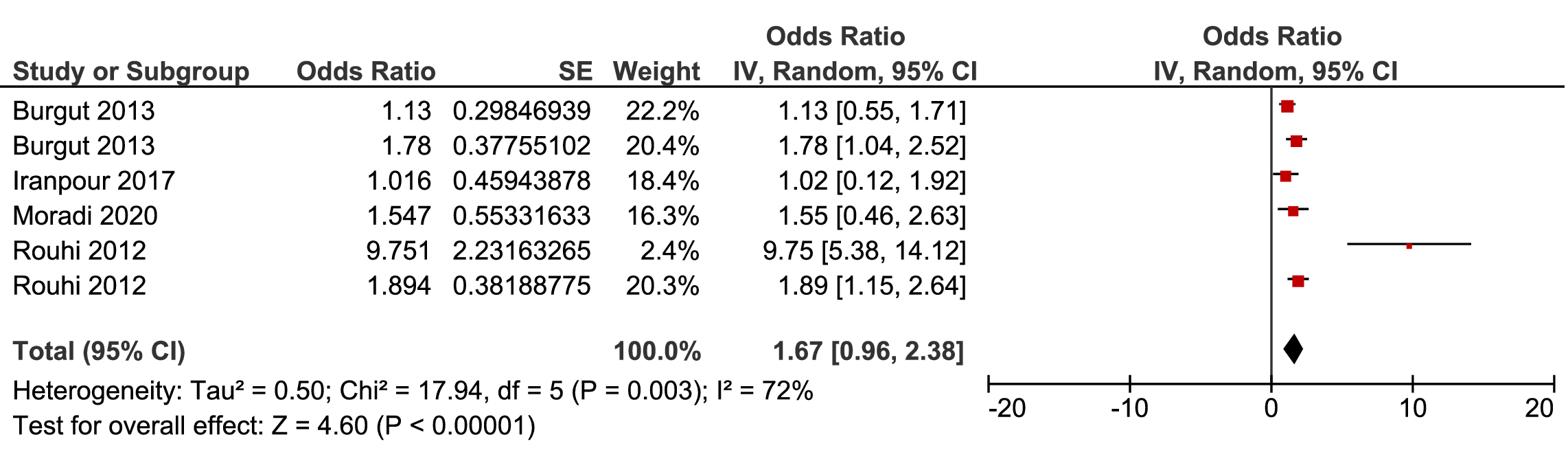


**Additional figure 10:** Forest plot for pooled association between housewife (Ref. Mother’s occupation) and postpartum depression in the Middle East.


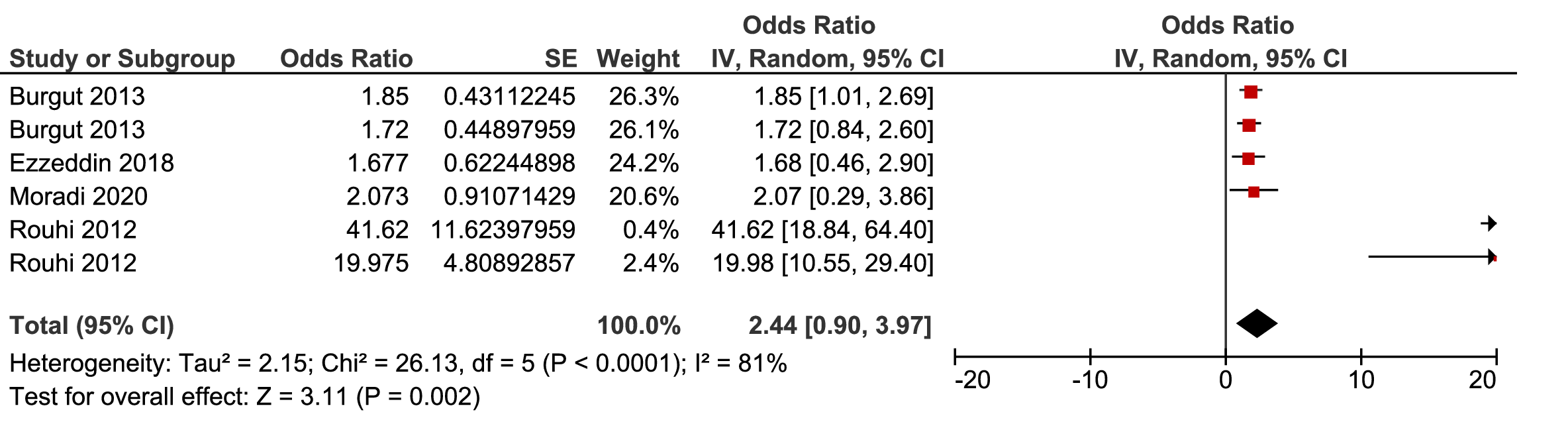


**Additional figure 11:** Forest plot for pooled association between unplanned pregnancy (Ref.Planned pregnancy) and postpartum depression in the Middle East.


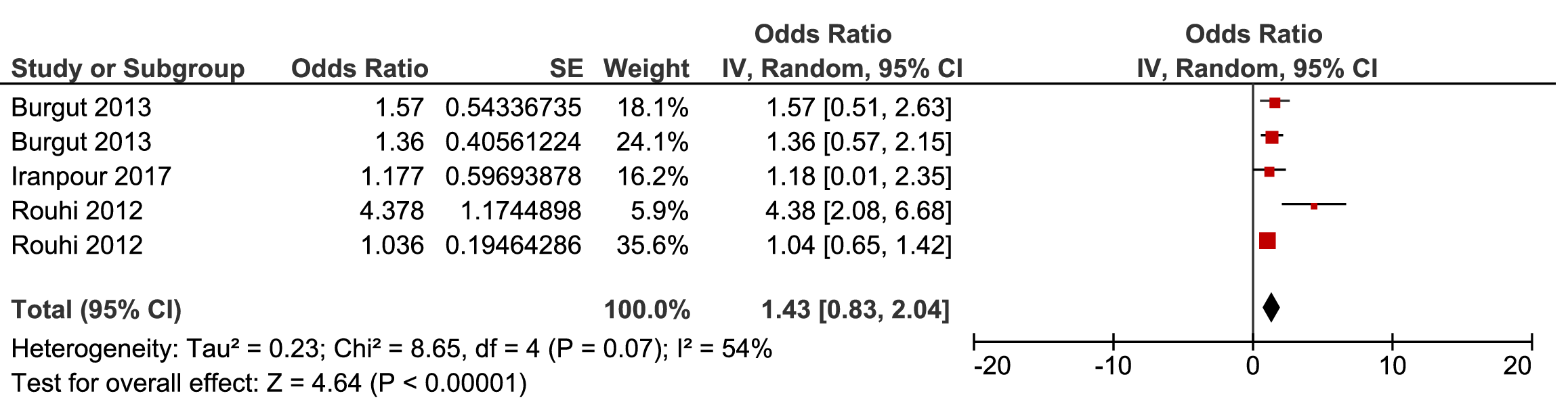


**Additional figure 12:** Forest plot for pooled association between young age (Ref. Age) and postpartum depression in the Middle East.


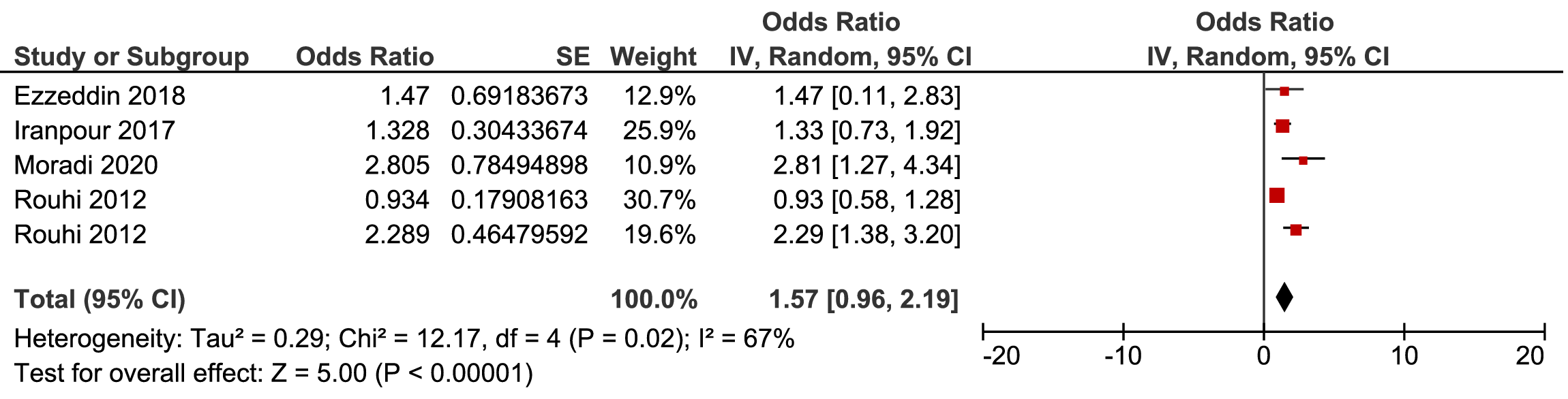


**Additional figure 13**: Forest plot for pooled association between unwanted baby gender (Ref.Baby’s gender) and postpartum depression in the Middle East.


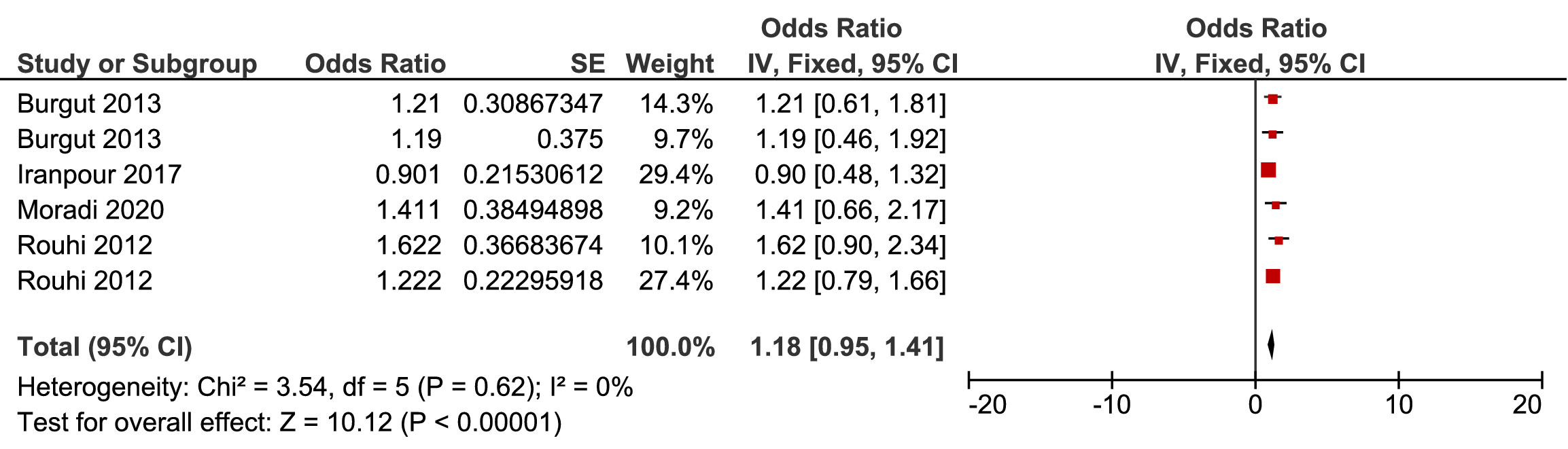


**Additional figure 14:** Forest plot for pooled association between mode of delivery and postpartum depression in the Middle East.


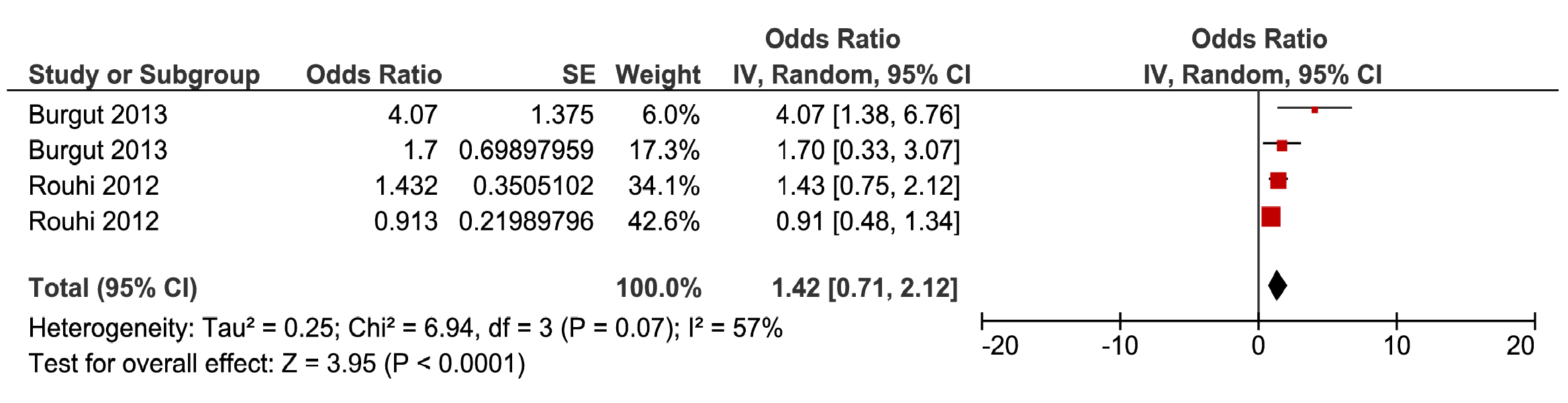


**Additional figure 15:** Forest plot for pooled association between feeding by formula (Ref. Type of feeding) and postpartum depression in the Middle East.
